# A digitalized analysis of incisal changes among orthodontically treated patients: A retrospective comparative study

**DOI:** 10.12688/f1000research.145095.1

**Published:** 2024-04-23

**Authors:** Nancy Ajwa, Alhanouf Binsaeed, Shaikhah Aloud, Raneem Alanazi, Hind Bin Mlafakh, Dalal Alajmi

**Affiliations:** 1 Preventive Dentistry Department, Riyadh Elem University, Riyadh, Saudi Arabia; 2 Dental intern, College of Medicine and Dentistry, Riyadh Elm University, Ryadh, Saudi Arabia

**Keywords:** Clear Aligner, Orthodontics, Metal brackets, Incisors Analysis, Inclination, Lateral Cephalometries

## Abstract

**Background:**

To compare incisor angulation and/or position changes among orthodontically treated patients with metal brackets and clear aligners.

**Methods:**

A total of sixty-two participants of both sexes, aged–16–40 years old, with CL I skeletal pattern and mild crowding following strict eligibility criteria were included. The patients were divided into two groups based on their treatment approach. Pre and post lateral cephalograms were collected from Riyadh Elm University (REU) and then digitally analyzed using WEBCEPH (Medical Image Analysis) software. Eight angular and two linear measurements were used for the assessment.

**Results:**

The upper incisor angulation and position showed statistically significant differences when orthodontic clear aligners were used. In contrast, no significant difference was observed with the conventional orthodontic treatment. However, the upper incisal palatal root torque decreased after clear aligner therapy compared to conventional treatment. The inter-incisal angle demonstrated a significant increase with clear aligners compared to conventional treatment.

**Conclusions:**

The current study revealed the importance of definitive guidelines upon and after treatment, in addition to determining incisor changes. Orthodontic clear aligners are distinct from conventional treatments in controlling the incisors’ angulation and position. The expansion treatment modality precedes Interproximal reduction in increasing the arch perimeter.

## Introduction

The scope for orthodontic treatment in creating beautiful smiles appears to be seamless, as people increasingly desire to be more conscious about their socially desirable appearance. Several parameters are considered while planning orthodontic treatment to recreate smiles, such as the smile arc, smile line, smile symmetry, and buccal corridors. ^1^ The development of digital imaging techniques and technologies has drastically improved over time, leading to the development of significantly superior aids in diagnostic and treatment planning, particularly in the field of orthodontics.

Among these, the incisal position in the arch is key to diagnosis. The angulation and inclination of crowns of teeth are said to affect the arch perimeter during orthodontic treatment planning. Therefore, incisal angulation plays an important role in supporting dentition and obtaining normal occlusion with facial harmony to the surrounding soft tissue. ^2^

Dental crowding, also known as swarming, especially in the anterior region, is one of the most common findings in orthodontic patients. ^3^ Dental crowding can be defined as a disparity in the relationship between tooth and jaw sizes, which results in imbrication and rotation of teeth. Dental crowding may occur because of excessively large teeth, excessively small bony bases of the jaws, and a combination of large teeth and small jaws. ^4^ The clinical forms of dental crowding may be classified based on the degree of manifestation: mild, moderate, or severe; location, anterior, intermediary, lateral, or posterior position; and cause/etiology: primary, secondary, tertiary, combined, or transient. ^5^

Transverse arch expansion, tooth extraction, and interproximal enamel reduction (IPR) are among the several methods suggested to relieve dental crowding. ^3^ When arch widths are less in the case of crowding when compared to spacing, this is an indication of arch expansion to accommodate all teeth. ^6^ Extraction of all first premolars is usually indicated in cases of moderate to severe crowding in the labial segment to create space and relieve crowding. ^7^ Finally, IPR is a clinical procedure that involves reduction, anatomic re-contouring, and protection of the interproximal enamel surfaces of permanent teeth, and it is indicated for patients with mild to moderate crowding (4-8 mm), which is an alternative procedure to extraction. ^8^

Orthodontic treatment may require tooth extraction to create space for teeth to move into their newer, straighter positions. Owing to the expected effect on the dental arch length, there has been a continuous debate between extraction and non-extraction planning. ^9^ A study conducted by Konstantonis ^10^ compared the difference between both protocols, and the results showed a significant increase in lip retraction alongside the development of the morea obtuse nasolabial angle among the extraction subjects. ^11^ However, incisor display was insignificant. Orthodontic diagnosis and treatment planning on accurately analyzed lateral cephalometric radiographs. ^10^ Although cephalometric studies are subject to errors and are mostly affected by bias, randomization of measurements is one of the most effective methods for avoiding bias. ^12^ However, metal brackets have been the conventional and effective orthodontic appliance for over a hundred years. Owing to the increase in patients’ demand for a more esthetic and comfortable orthodontic treatment, the technique has fueled the concerns of clear aligners. With the development of orthodontics, clear aligner therapy was introduced in the early 1900s, and the initial cases involved minor crowding or spacing. ^13^

Although both clear aligners alongside the metal brackets are effective in treating malocclusion, clear aligners have an advantage in the segmented movement of teeth and shorten the treatment duration. Moreover, metal brackets are more effective than aligners in achieving great improvement, producing adequate occlusal contacts, controlling teeth torque, and increasing transverse width and retention. Therefore, clinicians should consider the characteristics of these orthodontic appliances when making treatment decisions. ^13^ Therefore, the rationale of the current study was to assess the efficiency of clear aligners’ expansion mechanism of action and determine if it would influence the need for IPR in overcoming incisal flaring and/or crowding compared with conventional fixed appliances. Hence, this study aimed to compare incisors’ angulation and/or position changes among orthodontically treated subjects with metal brackets versus clear aligners.

## Methods

### Design

A retrospective comparative study was designed which utilized pre- and post-cephalometric radiographs that were digitally analyzed using WEBCEPH (Medical Image Analysis) software version 1.5.0 ^14^ An open access alternative is Forabi/WebCeph2.

### Sample size estimation

A sample size of 62 was determined using G* Power version 3.9 software. The effect size was estimated based on previous assessments of incisor inclination in Class I skeletal adult patients.

### Study population

The mean age of the patients included in the study was 16–40 years old. The inclusion and exclusion criteria are listed in Table 1. The participants were divided into the following two groups:

**Table 1.** Subjects’ eligibility criteria.

Inclusion criteria	Exclusion criteria
Class I skeletal and dental patterns	Class III or Class II skeletal or dental patterns
Patient with Mild crowding ≤4 mm	Patient with Moderate to Sever crowding (5 mm or more)
Non-extraction cases	Patient with anterior prosthesis
+ve overjet (less than 4 mm)	Patient with history of ortho or interceptive treatment
No extensive tooth decay	-ve overjet
Both genders	Patient with congenitally missing interiors
	Patients with systemic disease that would comprise the periodontium
	Patients with history of craniofacial trauma, surgery, TMJ or orofacial pain

TMJ: Tempero Mandibular Joint.

**G1:** treated by clear aligners (Invisalign)

**G2:** treated by conventional metal brackets

### Ethical statement

The study (Approval number: “FUGRP/2022/278/814/783”, dated 20 August 2022) was conducted in full compliance with the Institutional Review Board at Riyadh Elm University (REU) Research and Innovation Center and the study commenced after obtaining approval from the Board. Written informed consent was obtained from the patients before starting the procedure.

### Data collection

Data for the study were collected between October and November 2022 and attained a convenient sample of (n = 65) files, each having pre- and post-lateral cephalometric radiographs. Of these, 62 files were included in the study to equalize both groups (n = 31 in each group). The included radiographs were collected from orthodontic clinics at Riyadh Elm University (REU) alongside multiple private practices in Riyadh, Saudi Arabia. A pilot study and reliability tracing were conducted between the investigators. Inter-examiner reliability was determined using Cohen’s Kappa statistics, and a score of K = 0.825 was achieved, indicating adequate reliability, which allows for minimum measurement errors. Radiographs selected for the pilot study were not included in the final study sample.

### Instruments

**Figure 1.**
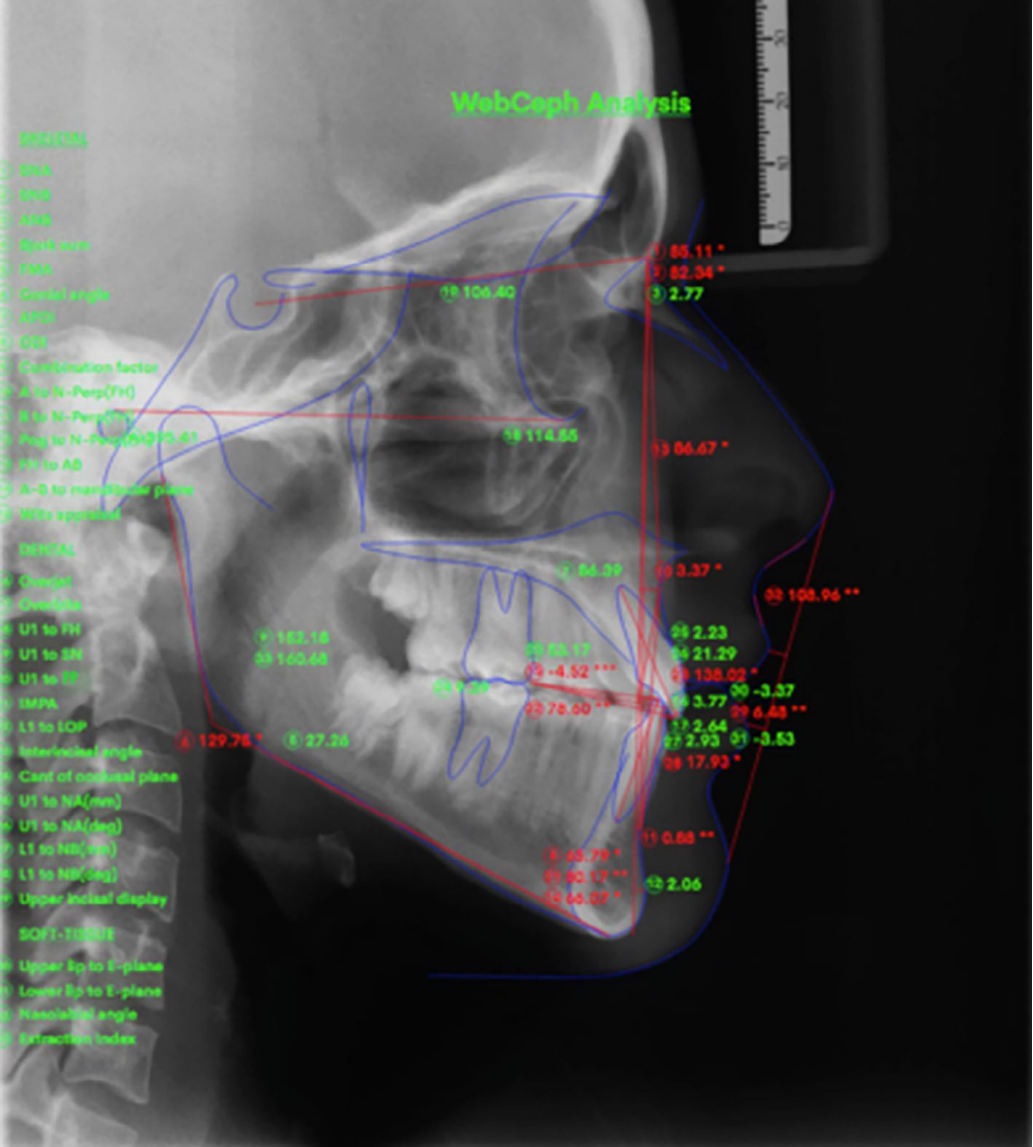
WEBCEPH analysis of linear and angular measurements.

Digital tracing of the enrolled lateral cephalometric radiographs was conducted at T0 and T1. Innovative artificial intelligence software (WEBCEPH Medical Image Analysis, Version 1.5.0) designed and coded by an orthodontist was used. The software was used to fabricate analysis of certain angles alongside linear interpretations in reference to the measurements as described in Figure 1 and Table 2. ^15^ Double confirmation of repeated cephalometric tracing was performed to reduce the potential number of errors within the analysis.

**Table 2.** Lateral cephalometric measurements and interpretation description.

Measurement	Type	Description
SNA	*Angular*	Angle between lines SN and NA representing the position of Max in relation to the cranial base
SNB	*Angular*	Angle between lines SN and NB representing the position of Mand in relation to the cranial base
ANB	*Angular*	Angle between lines AN and NB representing the skeletal classification
U1-SN	*Angular*	Angle between a line through the long axis of the upper central incisor and SN line represents the UI angulation to the cranial base
UI-FH	*Angular*	Upper incisor to Frankfurt horizontal plane represents the UI angulation
UI-PP	*Angular*	Upper incisor to palatal plane represents the UI angulation
UI-NA	*Angular*	Angular Angle between a line through the long axis of upper central incisor and NA line
LI-MPA	*Angular*	Formed by the mandibular plane and a line drawn down the long axis of the mandibular incisor representing the LI angulation
LI-NB	*Angular*	Angular Angle between a line through the long axis of lower incisor and NB line
UI-LI	*Angular*	Angle between a line through the long axis of upper and lower incisors representing the inter-incisal angle
Overjet	*Linear*	Horizontal distance between tips of upper incisor and the labial surface of lower incisor
Wits Value	*Linear*	Measurements of perpendicular projection of points A and B to occlusal plane to confirm the classification

SNA: (Sella, Nasion A point), SNB: (Sella, Nasion B point), ANB: Angels between (A point, Nasion B point), UI: (Upper incisor), PP (palatal plane), MPA: (Mandibular Plane Angle), LI: (Lower incisor), NA: (Nasion A point).

For both groups, the required variables were gender, age, whether conventional or clear aligners were used, and whether expansion or IPR was performed. For the clear aligners group, the required information was collected from private practices in Riyadh, alongside the 3-dimensional virtual simulation (Clin-check) of an orthodontist prescribed that shows the stages of the treatment plan and makes the required changes observed for each case, respectively.

Clin-check enables clinicians to make changes to the treatment plan themselves, such as changing the size and rotation of the attachments. The arch shape can also be adjusted as needed by expanding or narrowing the anterior or posterior segments of the aligners. It has specific tables that prescribe the required amount of IPR and its position, whether anterior or posterior, in the upper or lower arches. In addition, it also provides expansion details that affect the intercanine or intermolar width, reflecting a glance over expected changes throughout the patient’s treatment journey. On the other hand, for conventional metal cases, the site of IPR and/or type of expansion using an appliance or an archwire were collected and exported from the included patient’s files (progress notes) written by his/her orthodontists.

Descriptive statistics of the frequency distribution, percentages, means, and standard deviations were calculated for the study variables. The study data followed a normal distribution. Pre and post changes in cephalometric variables after conventional orthodontic treatment and clear aligner therapy were compared using paired t-tests. At the same time, an independent samples t-test was applied to compare the mean differences in cephalometric variables between conventional and clear aligner orthodontic treatments. Differences were considered statistically significant at P <0.05. Data analyses were performed using SPSS version 25 (IBM-SPSS, Armonk, NY, USA).

## Results

Table 3 shows the distribution of the study variables. A total of 31 patients who underwent fixed orthodontic therapy using the conventional method and 31 who used clear aligners were included in this study. Most study participants were females in both treatment groups. Irrespective of whether the patients were treated using the conventional method or clear aligners, expansion, IPR, or both were performed on these patients. However, more than half (54.8%) of the clear aligner patients received IPR.

**Table 3.** Distribution of study variables.

Variables		Conventional		Clear aligner	
		n	%	n	%
**Gender**	Male	8	25.8%	13	41.9%
	Female	23	74.2%	18	58.1%
	Total	31	100.0%	31	100.0%
**Expansion/IPR**	Expansion	14	45.2%	16	51.6%
	IPR	17	54.8%	10	32.3%
	Both	0	0.0%	5	16.1%
	Total	31	100.0%	31	100.0%
**Age in years (Mean ± SD)**		28.97 ± 4.35		18.81 ± 2.57	

IPR: (Interproximal Reduction).

The mean age of the patients treated by the conventional method was 28.97 ± 4.35 years, while for the patients treated by clear aligners was found to be 18.81 ± 2.57 years. The mean and standard deviation values of each cephalometric variable before and after the conventional orthodontic treatment are shown in Figure 1. All variables considered in this study showed slight changes in mean values pre and after treatment.

**Figure 2.**
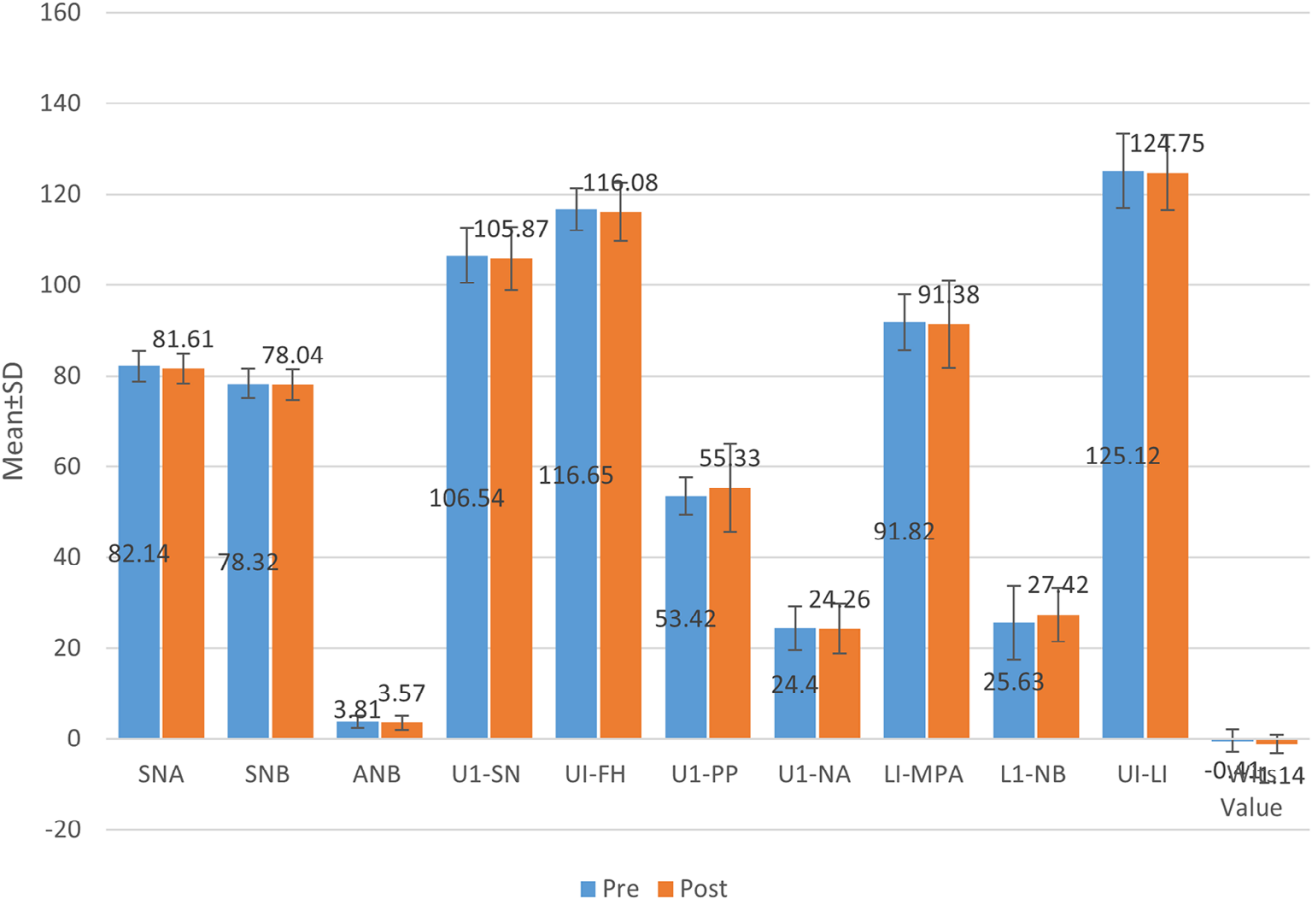
Conventional orthodontic treatment.

**Figure 3.**
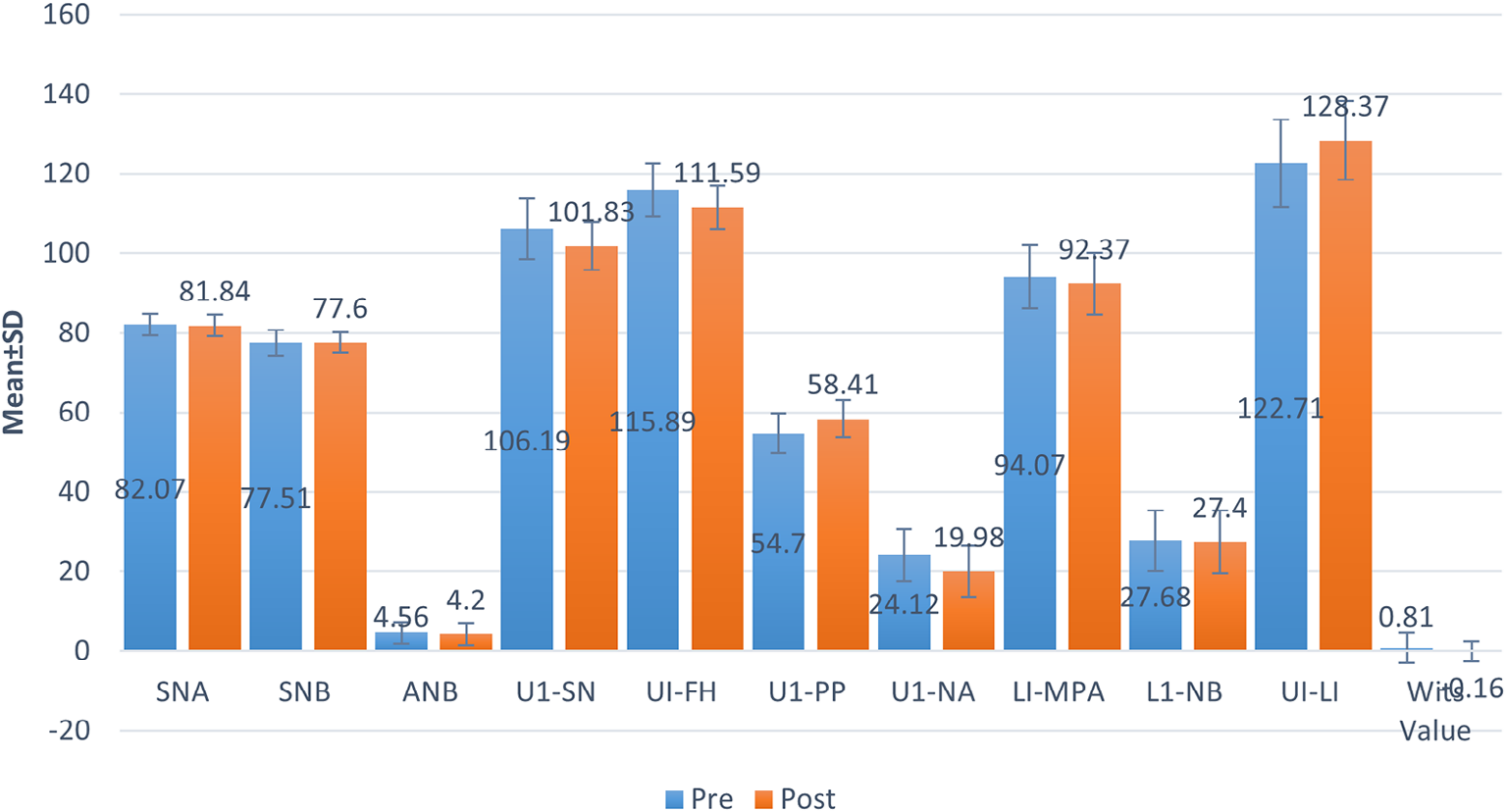
Clear aligner treatment.

Figure 2 shows the pre- and post-treatment changes in cephalometric variables following clear aligner orthodontic treatment. Cephalometric variables of U1-SN (106.19 ± 7.57 vs.101.83 ± 5.97, p = 0.001), UI-FH (115.89 ± 6.74 vs. 111.59 ± 5.40, p < 0.001), U1-PP (54.70 ± 5.01 vs. 58.41 ± 4.66, p = 0.002), U1-NA (24.12 ± 6.64 vs. 19.98 ± 6.38, p = 0.002), UI-LI (122.71 ± 10.93 vs. 128.3 ± 79.95, p = 0.005) showed statistically significant difference before and after treatment using Clear aligners. In contrast, no such significance was observed with conventional orthodontic treatment using paired t-test ( Table 4 & Figure 3).

**Table 4.** Pre- and post-changes in cephalometric variables after conventional and clear aligner treatment.

Variables	Conventional					Clear aligner				
	Pre		Post		*p* *^¶^*	Pre		Post		*p* *^¶^*
	Mean	SD	Mean	SD		Mean	SD	Mean	SD	
**SNA**	82.14	3.34	81.61	3.32	0.070	82.07	2.67	81.84	2.72	0.515
**SNB**	78.32	3.21	78.04	3.34	0.316	77.51	3.14	77.60	2.60	0.743
**ANB**	3.81	1.42	3.57	1.56	0.295	4.56	2.76	4.20	2.81	0.211
**U1-SN**	106.54	6.05	105.87	6.98	0.491	106.19	7.57	101.83	5.97	***0.001*** *
**UI-FH**	116.65	4.57	116.08	6.42	0.563	115.89	6.74	111.59	5.40	***0.000*** *
**U1-PP**	53.42	4.17	55.33	9.70	0.257	54.70	5.01	58.41	4.66	***0.002*** *
**U1-NA**	24.40	4.84	24.26	5.49	0.881	24.12	6.64	19.98	6.38	***0.002*** *
**LI-MPA**	91.82	6.21	91.38	9.62	0.770	94.07	7.98	92.37	7.73	0.199
**L1-NB**	25.63	8.19	27.42	5.94	0.205	27.68	7.67	27.40	8.02	0.844
**UI-LI**	125.12	8.20	124.75	8.30	0.829	122.71	10.93	128.37	9.95	***0.005*** *
**Wits Value**	-0.41	2.46	-1.14	2.01	0.088	0.81	3.81	-0.16	2.52	0.081

SNA: (Sella, Nasion A point), SNB: (Sella, Nasion B point), ANB: Angels between (A point, Nasion B point), UI: (Upper incisor), PP (palatal plane), MPA: (Mandibular Plane Angle), LI: (Lower incisor), NA: (Nasion A point).

^¶^ Paired t-test.

* Significant at 0.05.

A comparison of the mean differences in cephalometric variables between clear aligner therapy and conventional orthodontic treatment using an independent t-test is shown in Table 5. Cephalometric variables UI-SN (-4.361 ± 6.770 vs. -0.670 ± 5.348, p = 0.020), UI-FH (-4.301 ± 5.727 vs. -0.574 ± 5.461, p = 0.011), and UI-NA (-4.135 ± 6.612 vs. -0.141 ± 5.183, p = 0.010) were found to be significantly decreased in torque after clear aligner therapy compared with conventional orthodontic treatment. However, UI-LI (5.660 ± 10.279 vs.-0.369 ± 9.398, p = 0.019) demonstrated a significant increase with clear aligner therapy compared with conventional treatment. The intercanine and intermolar widths were significantly higher in the expansion cases than in the IPR cases (p < 0.05) ( Table 6). The amount of orthodontic space gained was not significantly higher in the upper jaws than in the lower jaws after IPR ( Table 7).

**Table 5.** Comparison of mean differences of cephalometric variables treatment.

Groups		Mean	SD	SEM	t	*p* *
**SNA**	Clear aligner	-0.227	1.924	0.346	0.680	0.499
	Conventional	-0.531	1.571	0.282		
**SNB**	Clear aligner	0.091	1.537	0.276	0.960	0.341
	Conventional	-0.288	1.574	0.283		
**ANB**	Clear aligner	-0.356	1.551	0.279	-0.325	0.746
	Conventional	-0.240	1.253	0.225		
**UI-SN**	**Clear aligner**	**-4.361**	**6.770**	**1.216**	**-2.382**	***0.020***
	Conventional	-0.670	5.348	0.961		
**UI-FH**	**Clear aligner**	**-4.301**	**5.727**	**1.029**	**-2.623**	***0.011***
	Conventional	-0.574	5.461	0.981		
**UI-PP**	Clear aligner	3.715	6.075	1.091	0.907	0.368
	Conventional	1.914	9.233	1.658		
**UI-NA**	**Clear aligner**	**-4.135**	**6.612**	**1.187**	**-2.647**	***0.010***
	Conventional	-0.141	5.183	0.931		
**LI-MPA**	Clear aligner	-1.697	7.197	1.293	-0.647	0.520
	Conventional	-0.432	8.171	1.467		
**LI-NB**	Clear aligner	-0.283	7.958	1.429	-1.042	0.301
	Conventional	1.788	7.687	1.381		
**UI-LI**	**Clear aligner**	**5.660**	**10.279**	**1.846**	**2.410**	***0.019***
	Conventional	-0.369	9.398	1.688		
**WITS**	Clear aligner	-0.965	2.976	0.535	-0.340	0.735
	Conventional	-0.735	2.317	0.416		

SNA: (Sella, Nasion A point), SNB: (Sella, Nasion B point), ANB: Angels between (A point, Nasion B point), UI: (Upper incisor), PP (palatal plane), MPA: (Mandibular Plane Angle), LI: (Lower incisor), NA: (Nasion A point).

* Independent t-test.

**Table 6.** Comparison of the mean of changes between expansion and IPR.

Region	Expansion/IPR	N	Mean	SD	t	df	p
Amount of expansion (U Inter-Canine)	Expansion	14	.8214	.62163	4.629	14.176	***0.000***
	IPR	17	.0353	.14552			
Amount of expansion (U Inter-Molar)	Expansion	14	1.1643	1.20167	3.511	13.246	***0.004***
	IPR	16	.0313	.12500			
Amount of expansion (L Inter-Canine)	Expansion	14	.7286	.60945	2.858	28	***0.008***
	IPR	16	.1688	.46147			
Amount of expansion (L Inter-Molar)	Expansion	14	.5786	.55217	3.822	13.187	***0.002***
	IPR	16	.0125	.05000			

IPR: (Interproximal Reduction).

**Table 7.** Amount of change in upper and lower arches.

Jaw	N	Mean	SD	df	t	p
Upper	31	1.0355	1.11551	0.565	60	0.574
Lower	31	.8806	1.04257			

## Discussion

The Assessment of incisor inclination is a major part of orthodontic treatment. The main objective of this study was to compare the effects of metal brackets and clear aligners on changes in incisor position. By choosing cephalometric landmarks and based on their predictability, the results were predictable, and the use of the WEBCEPH for the calculations reduced measurement errors. ^14^^,^^16^^,^^17^

Clear aligner patients were significantly younger than those in the fixed appliance group (18.81 and 28.97 years old, respectively), which was indicative of younger patients preferring clear aligners over the conventional method for esthetic benefit, which upholds their level of confidence. However, the findings of our study contrasted with those of a study conducted in Ohio by Gu et. al., in which older patients underwent orthodontic correction by Invisalign. The contrasting result could be suggestive of the consciousness regarding physical appearance, easier approach to treatment by placing the trays in the mouth as directed by orthodontists, and shorter treatment time with clear aligners. ^18^ Since all patients included in the current study were older than 16 years, it was unlikely that growth played a significant role in the treatment outcome.

Our study included more female patients than male patients in both groups, which holds true because females are more concerned about their aesthetics and appearance. ^19^ Clements *et al*. conducted a study at the University of Washington. al. supported clear aligners as being more successful in improving anterior alignment, transverse relationships, and overbite than fixed appliances, indicating a significant reduction in UI-SN, UI-FH, and UI-NA, because clear aligners improved the anterior segment by lowering upper incisor flaring, which is consistent with our study findings. The study also suggested that clear aligners have no adverse effects on gingival health during treatment. ^20^

A study by Ke *et al.* compared the treatment effectiveness between fixed orthodontic treatment and clear aligners and found that tooth torque was controlled more effectively when fixed appliances were used ^13^; however, our study showed that clear aligners were more effective in controlling incisor inclination and showed a statistically significant difference in the inter-incisal angle (UI-LI) before and after treatment. Hence, we reject the null hypothesis and state the alternative hypothesis that clear aligners are more effective than conventional fixed appliances in improving incisor inclination.

A study by Kassas *et al*. at the State University of New York evaluated 31 Invisalign cases before and after treatment and the results of their study showed a significant improvement between the pre- and post-treatment models of the patients which supports our hypothesis that clear aligners provide a useful orthodontic correction in cases of mild to moderate malocclusions. ^21^ However, the retrospective study by Djue *et al*. compared the outcomes of Invisalign treatment to those of fixed appliances using the objective grading system of the American Board of Orthodontics (ABO) in a sample of adult patients and showed that treatment with fixed appliances was significantly more effective than treatment with Invisalign, which was the least successful in correcting occlusal contacts. ^22^

In relation to existing literature on incisal inclination, Ghaleb *et al.* evaluated the impact of maxillary incisor inclination on the aesthetics of the profile view of a smile and found that smile aesthetics in the profile view were dramatically affected by incisor inclination. ^23^ Cao *et al.* reported that maxillary incisor protrusion and lingual inclination were preferable to retruded or flared incisors, although increased maxillary incisor labial inclination might flatten the smile arc and decrease the incisal display, which showed the importance of aesthetics and appearance in the current study. ^24^

The use of artificial intelligence in the WEBCEPH software for analyzing cephalometric radiographs leads to absolute measurements. Katyal *et al.* in their study, WEBCHEPH was more reliable than digital tracing using FACAD and manual tracing for analyzing lateral cephalometric radiographs. Moreover, multiple other studies conducted over various other imaging software to evaluate the incisor inclination in lateral cephalometries such as Aldrees, ^25^ used Dolphin Imaging ^®^ 10.0 software. ^26^ Shahakbari *et al.* who used Onyx Ceph version 3.6 software examined its reliability compared to manual measurements. ^27^

IPR and expansion are frequently used methods in conjunction with orthodontic treatments, and this has gained popularity in view of the increasing trend towards non-extraction-based treatment modalities. ^28^ In the current study, the amount of expansion was compared with the IPR performed. It was found that the inter-canine and inter-molar distances of both upper and lower arches were significantly higher in expansion in comparison to the IPR cases, thus providing more space to facilitate treatment and improve the final results, which was in line with a study conducted by Chung *et al.* in 2015, which suggested that expansion is the treatment of choice in the presence of a normal-sized dentition and decreased arch width because it enables widening of the dental arches, which predictably increases the arch perimeter, which could be a reason for the expansion method to create more space than IPR. ^29^

Our study has certain limitations. Since the design of our study was retrospective, some difficulties were encountered in tracing the exact amount of IPR and expansion values among the fixed appliance group due to insufficient documentation. The data that support the findings of this study are openly available in public repository. ^30^

## Conclusions

Within the limitation of the current study, it can be concluded that:

• Definitive guidelines are needed to determine changes in incisal angulation upon and after treatment.
• The expansion treatment modality precedes IPR in increasing the arch perimeter.
• A clear orthodontic aligner is a preferable treatment modality to control the incisor’s angulation/position.
• The palatal root torque of the upper incisors decreases effectively when using clear aligners compared to conventional therapy, while the inter-incisor angle is sufficiently increased.

### Recommendations

1. An adequate number of subjects and parameters were included in this study; however, we recommend further studies with a larger number of subjects and cephalometric parameters to generalize the current findings and to be conducted at the population level.
2. A study that includes the use of ceramic brackets compared to other types of orthodontic appliances is also recommended.
3. Further comparison between WEBCEPH and other commonly used software, such as OnyxCeph and Dolphin, is also helpful to assess the accuracy of the general outcomes.

## Consent

Written informed consent for publication of their clinical details and clinical images was obtained from the patients.

## Data Availability

Figshare: Underlying data for ‘A digitalized analysis of incisal changes among orthodontically treated patients: A retrospective comparative study’, https://doi.org/10.6084/m9.figshare.24647484.v1. ^30^ Data are available under the terms of the Creative Commons Attribution 4.0 International license (CC-BY 4.0).
