# Supplementary material for: A digitalized analysis of incisal changes among orthodontically treated patients: A retrospective comparative study
Source: F1000Res. 2024 Apr 23;13:343. [Version 1] doi: 10.12688/f1000research.145095.1 (PMC11234081; doi:10.12688/f1000research.145095.1)
Supplement: Peer review history

# Reviewer report: Reviewer response for version 1

**DOI:** 10.5256/f1000research.158984.r298723

**Published:** 2024-07-09

**Authors:** Tiago Fialho

**Affiliations:** 1 University of São Paulo, Bauru, Brazil; 2 Ingá University Center UNINGÁ, Maringá, Brazil

This study aims to compare metal and Invisalign aligners regarding changes in the angulation or position of the incisors at the end of orthodontic treatment.

 The Summary is well written, faithfully summarizing what was studied.

 The Introduction provides a clear explanation of the study's purpose, giving the reader a good basis. There are discreet punctuation errors, which do not hinder reading or reduce the quality of the text.

 In the Methods section, I wished for a more detailed explanation about the sample size and how this number was achieved, but the rest of the text is well described. The tables and figures are very explanatory, providing details, and are easy for the reader to interpret.

 The results are meticulously explained, providing a comprehensive understanding of the study's objectives and outcomes.

 In the Discussion, although there is a good work of references in analogies with the present study, the results could have been explored further to emphasize what was observed. However, the text is clear and explains what the authors sought.

 The Conclusion, while acknowledging the study's limitations, effectively underscores the value of the results obtained, making a significant contribution to the field of orthodontics.

 General evaluation:

 Although it has limitations, this study is of great assistance to the orthodontist in choosing the orthodontic device to recommend in each case. It is especially important in cases of crowding and its resolution. The text is clear, easy to read, and interpret.

Is the work clearly and accurately presented and does it cite the current literature?

Yes

If applicable, is the statistical analysis and its interpretation appropriate?

Yes

Are all the source data underlying the results available to ensure full reproducibility?

Yes

Is the study design appropriate and is the work technically sound?

Yes

Are the conclusions drawn adequately supported by the results?

Yes

Are sufficient details of methods and analysis provided to allow replication by others?

Yes

Reviewer Expertise:

Orthodontics, Aligners

I confirm that I have read this submission and believe that I have an appropriate level of expertise to confirm that it is of an acceptable scientific standard.

# Reviewer report: Reviewer response for version 1

**DOI:** 10.5256/f1000research.158984.r284506

**Published:** 2024-06-19

**Authors:** Ashwin Shetty Yermal

**Affiliations:** 1 Imam Abdulrahman Bin Faisal University, Dammam, Eastern Province, Saudi Arabia

The study effectively compares incisor angulation and/or position changes among orthodontically treated patients with metal brackets and clear aligners.

 The abstract effectively summarizes the study's purpose, methods, results, and conclusions. It provides a clear snapshot of the study, highlighting the key findings comparing incisor angulation and/or position changes among orthodontically treated patients with metal brackets and clear aligners.

 The methods section is well-detailed, allowing for replication of the study. The retrospective design is appropriate for the research questions posed. The sample size is adequate.

 The results are presented clearly, with appropriate use of tables and figures to illustrate key findings. The statistical analyses are robust, and the results are interpreted correctly. The study effectively highlights comparison of incisor angulation and/or position changes among orthodontically treated patients with metal brackets and clear aligners.

 The discussion section provides a thorough interpretation of the results, linking them back to the research questions and hypotheses. The authors discuss the implications of their findings in the context of existing literature.

 Overall Assessment:

 This study provides valuable insights into comparison of incisor angulation and/or position changes among orthodontically treated patients with metal brackets and clear aligners. The methodology is sound, and the findings are clearly presented and interpreted.

Is the work clearly and accurately presented and does it cite the current literature?

Yes

If applicable, is the statistical analysis and its interpretation appropriate?

Yes

Are all the source data underlying the results available to ensure full reproducibility?

Yes

Is the study design appropriate and is the work technically sound?

Yes

Are the conclusions drawn adequately supported by the results?

Yes

Are sufficient details of methods and analysis provided to allow replication by others?

Yes

Reviewer Expertise:

Dental research

I confirm that I have read this submission and believe that I have an appropriate level of expertise to confirm that it is of an acceptable scientific standard.
